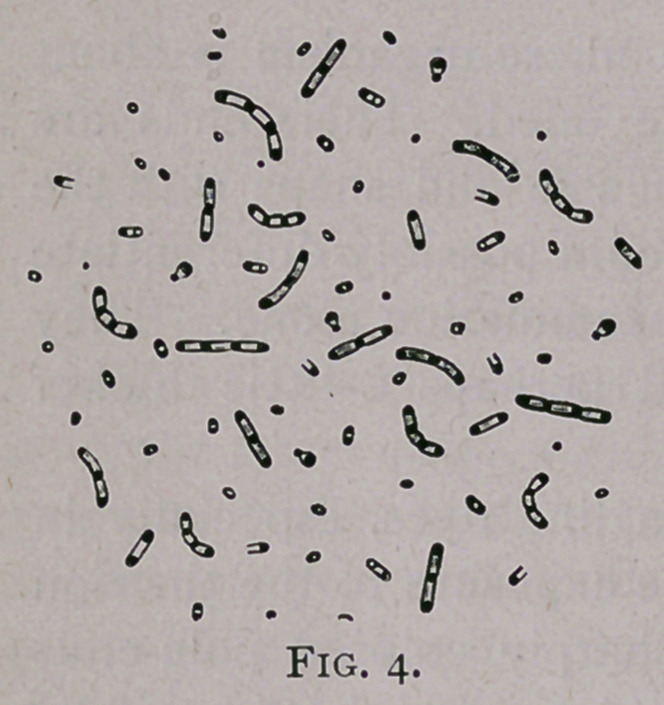# The Etiology of Southern Cattle Plague—Texas Fever

**Published:** 1892-09

**Authors:** Frank S. Billings

**Affiliations:** Director of the Patho-Biological Laboratory of the State University of Nebraska


### THE ETIOLOGY OF SOUTHERN CATTLE PLAGUE—TEXAS FEVER.

(*Continued*).

BY FRANK S. BILLINGS,

*Director of the Patho-Biological Laboratory of the State University of Nebraska.*

### THE BACILLUS OF SOUTHERN CATTLE PLAGUE PROVEN.

In the foregoing pages I have placed before the reader all the negative evidences, so far as I know, published by the investigators of the Government, against the validity of a specific bacillus being the cause of the "Texas Fever" in Cattle, as well as endeavored to show the utter unworthiness and unscientific character of this negative testimony. The reader must himself judge as to whether a case has been made out against the opponents of the investigations published from this Laboratory or not. I now enter on a much more pleasant task, that of placing the postive evidence resulting from quite a series of investigations before my readers.

While, I think, nearly every competent investigor will agree with me that we must use common sense and that the fulfilment of certain conditions, such as the invariable presence of one form of germ-life in every case of a given infectious disease, if the examinations are made at a time to obviate first, mortal or adventitious complications, still it is always well to fulfill the Koch conditions if we can, and happily we generally can in infectious diseases of animal life, at least as soon as we have learned how to provide suitable artificial cultivating media to the life-necessities of the germ discovered in the tissues of given animals affected with a specific infectious disease.

In regard to the bacillus of the *Southern Cattle Plague* I am absolutely certain that in my investigations every one of Koch's postulates have been as absolutely fulfilled as it is possible to do it, or has been done by him or any other investigator, in relation to any infectious disease of man or beast, the results of which are accepted by all competent men as conclusively proven. It is even negatively admitted by the chief government investigator that bacteria have been found in the blood and tissues of animals disdiseased with the "*Southern Cattle Plague*," though he denies their value, he says: "All the illustrations which have been published showing preparations of blood from Texas Fever Animals swarming with bacteria, *and sections showing the same micro-organisms*, are misleading and may be put aside as of no value to the student of this disease."

As has been said before, those are "bold words." The failure of one person in any given direction is no criterion by which to judge the success of another in the same direction. Were that so, the majority of scientific acquisitions would still be failures. It has been asserted, above, that this work on the "*Southern Cattle Plague*" fulfils the most exacting requisitions of modern bacteriological investigation. A much more unbiased and hence admittedly more honest opinion than that of the chief Government investigator has been expressed by Eulinberg in the latest edition of his "Bacteriologische Diagnostik," 1892, a standard work. There full credit is given us for the discovery of the bacillus of this disease, and it would not be given were the evidence not considered to be reliable and sufficient. *Not one European critic has ever questioned its sufficiency*. We have spoken of "Koch's postulates:" what are they? They are certain conditions which when fulfilled in the exact detail must necessarily lead to the acknowledgment of the discovery of a speeific disease-producing germ, as

1st. That in the tissues of animals diseased with a specific-infectious disease, a disease which has been long recognized by established clinical and miscroscopic phenomena; and when no secondary complications disturb the character of each case, and the animal is either killed or is examined before post-mortal changes can occur, *one and the same species of germ life invariably lie demonstrated.*

(*a*). By microscopic examination of the tissues of such animals.

(*b*). By cultivation in suitable artificial media, by inoculating the source from such tissues.

2d. That the same germ is found in the cultivations, and that on the inoculation of healthy individuals of the same species (not another) of animals from which the germ was obtained when ill with the disease in question, the same disease, in all its clinical and microscopical phenomena, is again produced.

3d. That in the tissues of the inoculated animal the same germ is found; that it is again cultivated from those tissues and shown by comparison to be the same, organized as that originally derived from an individual having the natural disease.

Now, I say, as I said in the two previous editions of this work, *every one of those conditions have been fulfilled in connection with the bacillus of the Southern Cattle Plague.* Can any one think for a moment that I or any other investigator would have boldness enough to face the verdict of the world with such a positive assertion, unless absolutely sure of his ground? It must not be forgotten, for a moment, that the "great objector" to the correctness of our investigations has been proven to be uncertain and unreliable in nearly every assertion that he has made regarding the specific bacillus of any specific disease in animals in this country, and with a persistency worthy of a nobler cause has condemned the work of nearly every other American investigator in the same line to be erroneous. Let us recapitulate a little:

In the Reports of the Department of Agriculture, 1878–1880, Dr. Detmers asserted a bacillus to be the cause of Swine Plague, and did give a description not only there, but in the "*American Naturalist*," by which any unprejudiced observer engaged in investigating the same disease can recognize the organism in the description. Finical objectors have asserted that Demters did not demonstrate his find by accurate culture and inoculations; that he did not fulfil the Koch postulates. That is true. Neither did Brauel or Pollender, the original discovers of Bacillus Anthracis, and yet the world does not deny them whatever honor there may be in having seen the organism first. Even Koch does not now insist on his postulates being so exactly fulfilled, for the discovery of the Influenza-bacillus has his endorsement. Where the diseases of man are concerned the postulates cannot always be fulfilled. Common sense must rule in scientific investigation as well as other things in daily life. Let me give a case which I have not published, and having no desire for priority, shall not mention in detail, as no practical benefits are furnished. There is a certain disease in animals in this country in what I can demonstrate one and the same germ in every freshly slaughtered case. I have reproduced the disease in the same species of animals by an inoculation with the fresh blood and organs rubbed up in heaters, and in smears from those animals demonstrated the same organism. *As yet I have absolutely failed in cultivating it.* Suppose I should publish a full description of the disease and its lesions and describe the germ as seen, and the results of such inoculations, would any reasonable investigator doubt the genuineness of the information? That is more than has been done with the influenza-bacillus and several other organisms the authenticity of which has not been much, if any, questioned.

The primary point in all such cases, where the chain of evidence is incomplete, is the character of the observer for competency and reliability. As investigator, his qualifications in those two directions are his "stock in trade." They are his "credit" in the bank of original investigation. Now I claim that my opponents have not yet succeeded in establishing a "line of credit."

Let us resume our capitulation, and we shall again see whether this is so or not.

In the reports of Agricultural Department, 1880–1884, the "head of the house" enters into a very energetic opposition to Detmer's "Bacillus suis," and claims that a "Micrococcus" is the only cause of Swine Plague. He even goes so far as to enter into a polemic defence of the discovery of this "Micrococcus" against the hypothetical claims of Pasteur, and tries to show that Klein (84) had demonstrated the same "Micrococcus" in the same disease.

In 1885, the same head says, "that he no longer considers a micrococcus to be the cause of all outbreaks of Swine Plague,', and though vain endeavors have been made to show identity between that micrococcus and the germ of the new swine-plague, unfortunately the only "micrococcus" described in 1885 "fluidified" gelatine, and we know nothing about the other one in that direction.

Then in 1885 the "head of the house," unlike Detmers, gave such a description and illustration of the germ of the true Swine Plague that its best acquainted friends would not be able to recognize that organism in the description.

In 1890 it is admitted that the germ of the true Swine Plague was not discovered until late in the Fall of 1885, which completely nullifies the "credit" of the "head" investigator's reports on the same subject from 1880 to 1885.

In 1886 the "head of the house" announced a new and wide-spread "Swine Plague" in the country, and yet, until to-day, the evidence in favor of the germ then discovered were causing a local specific disease is insufficient to establish "his credit" in the house of bacteriological investigation.

The "head of the house" asserts there is no grounds for assuming that the "*Corn-stalk Disease*" is caused by a germ, but the published evidence again disqualifies his "paper on the market."

The "head of the house" also put his "notes on the market," that a protozoon is the cause of the Texas Fever, but on account of totally insufficient endorsement they have not been accepted.

SOME USEFUL HINTS AS TO COLLECTING MATERIAL.

The description of the *Bacillus* of *Southern Cattle Plague* should be next in order, but before entering upon that task it seems well to give a short description of a few technical operations, which any practitioner can easily acquire, for the benefit of Veterinarians, especially those practicing in the stock-raising sections of the country. It very often happens that such Veterinarians desire the aid of laboratory investigation, and it is even more necessary, in the interests for which such laboratories as this have been and will be established, that the investigators attached to them have the aid of practitioners in different parts of the State, so that a simple practical method of obtaining cultures is also necessary. Such a method has been long in use here, and can be recommended to be as reliable as any exclusive laboratory method, and to do away with the necessity of carying artificial media into the field, if carried out in detail, as here explained.

Many practitioners undoubtedly desire to know how to make so minor investigations, and can supply themselves with a microscope, but have neither the time or the means to fit up a small laboratory. It has therefore been our endeavor to obviate the necessity of the latter to the greatest degree possible. The method here recommended is only applicable to facultative parasitic septicæmia diseases.

TOOLS.

One needs, aside from the tools necessary to open the animal, a common table-knife, and a small scalpel in a fixed handle ; the blade of a pocket knife can always be used, and a piece of platinum wire fixed in a glass-rod, or a steel knitting needle will do in an emergency. The easiest and most practical way of getting a flame to sterilize one's utensils with is to have a small tin salve box, filled with absorbent cotton and an ounce or two of alcohol in a bottle. Wet the cotton with alcohol and ignite it. This is much better than a spirit lamp, gives more flame, and is cheaper and more practical.

*As to opening a diseased animal and obtaining cultures from the same :*

Where possible, and in this western country it is nearly always so, in order to obtain pure cultures and obviate the probability of the presence of adventitious germs in the tissues of the animal, one should kill one in the earliest possible stage of the disease. This plan also has the advantage of presenting the initial and specific pathological lesions of the disease free from those secondary complications which often lead to mistaken conclusions as to its nature. For complete pathological studies recently deceased animals are better, but not for obtaining cultures.

The animal is placed on its back, and must be either fastened or held in that position. Then the skin is cut through along the middle of the abdomen from between the jaws to the anus and separated from the body, well down on the sides. In separating the fore-limbs from the walls of the thorax care must be taken not to cut across the great axillary vessels of the fore-arm, as all the blood is wanted in the animal not outside of it ; hence animals for this purpose should not be killed so as to bleed them to any extent, when it can as well be avoided.

*Blood-cultures, made direct from the animal :*

These are called "blood-cultures," because the already infested blood of the deceased animal is directly used as a cultivating medium. To this purpose one requires a few one ounce wide-mouthed glass-stoppered bottles, by which the stoppel fits over the neck of the bottle like a cap, instead of slipping into it. (The convenience of these bottles in work of this kind cannot be over-estimated, and every laboratory should have a supply. They can be easily sent to country practitioners, with directions how to use them, and are equally valuable for personal investigations). In the place of scent bottles the practitioner CAN use any ordinary small wide-mouthed cork-stoppered bottle, though it cannot be relied upon for satisfactory results to any such degree as the others. In either case the bottles must be first sterilized. The best and most practical way open to the practitioner is to fill the bottles partly full of cold water and put in the corks, not too tight, then put them into a vessel with cold water, and slowly heat to boiling; this will seldom injure the bottles, if made of good glass; after boiling half an hour, take out the bottles, pour out the water, while hot, and immediately fill them with alcohol; the cork-stoppered ones should be covered with thin paper, tied on. They can then be set aside, and are always ready for use. The fore-leg is then held at such a distance from the body as to extend the axillary vessels, and the tissues around the latter carefully cut away so as to expose the axillary vein. If the wind does not blow so hard as to make the use of the alcohol flame impossible, saturate the cotton in the tin box and ignite it, and sterilize the outer walls of the vein by gentle application of the flame; too much heat will coagulate the blood in the vessel. Another method, and one which must be resorted to when the wind blows hard, is to saturate some absorbent cotton with corosive-sublimate solution—1 to 500 aqua, which one should always have with him, and then wrap the vein with such saturated cotton for a few moments. The alcohol is next poured out of the bottles, and it is a good plan to touch a match to the remainder, holding the inside of the cork or stopple to the flame. As soon as it goes out close the bottle. Any little amount of alcohol remaining in the bottle, if all turned out, will not interfere with the result however. Now take your scalpel, and sterilize the blade in the flame, or when impossible on account of wind, in the sublimate solution (which will ruin that knife, however, but that does not matter), and remove the sublimated cotton, if used, from the vein, and cut through its, then, lower wall, making a gaping wound; hold the mouth of your bottle next to it and it will fill sufficiently as quickly as one can make a transfer with rods, as usually done. *Close the bottle at once.* Much experience has tanght me that "blood cultures" made in this way can not only be relied on but have this advantage, that even when the lege-artis cultures are made from the heart's blood and turn out negative from the scarcity of the desired organism in that tissue, that in a few days one will obtain magnificent cultures in these "blood-cultures," and if the tube cultures are also made as controls, the blood cultures will be found equally reliable. I was gradually led to this method from the trouble I had with the severe winds in the west, as in the majority of cases in studying herd diseases here the animals are so distant from the house, no barns are present, that one can find no protection for a flame. Again, it practically turns out that the danger of getting adventitious flora (fungi) into these blood cultures under such circumstances is less than into tubes. It is, or should be, self evident that only germs present in the blood will develop in it afterwards, if the bottles are sterile and no accidental germs blow in.

The same bottles can be used for putting pieces of organs in, but in septicaemic diseases the lymph-glands are the most available organs, and those of the inginual region the most favorably situated. The way to treat them is to carefully dissect out a whole gland, or if too large, a suitable end, then heat the outside of the gland in the flame, or if not convenient, drop into the corrosive sublimate solution a few moments, and then place it in one of the sterilized bottles. If there are no pollutions in the gland and all the germs have been killed on the outside, then the specific germ will go on developing within it. I have kept such glands and such blood several weeks in a thermostast, and got nothing but pure cultures out of them, though tested by plate cultures.

The next thing to do is to expose the internal organs, which we do by cutting a longitudinal section along the median line, from anterior to the sternum to the anus, taking care not to open the intestines. The covering of the abdominal cavity is then cut crossways from this cut, anterior to the external angle of the ilium, and from the longitudinal cut nearly down to the external ends of the lumbar-vertebræ ; then the same covering is cut away from its attachments to the last ribs ; the thorax is opened by cutting the ribs through on both sides of the sternum, about three inches above it, the sternum is then freed from its diaphragm attachment and clapped back towards the head on the neck. As we have already made "blood-cultures" from the axillary vein, we need not have recourse to the heart's blood in this case. But as the investigator should also make use of the heart's blood by inoculating tubes, when he can, and the same method of treatment is applicable to the other organs ; we will briefly sketch how the heart and those organs are to be treated. The first thing is to expose the right ventricle of the heart, which is done by first opening the pericardium and slipping it back, then the "table-knife" is heated, and the outside wall of the right ventricle is seared thoroughly with the red-hot knife; this being done, the scalpel is next heated hot, and the walls of the ventricle cut and burnt through with one rapid movement. With the heated and sterilized wire, cooled first, the different media are then inoculated by dipping the wire loop quickly into the blood in the ventricle, and touching the surface of, or dipping it quickly into the cultivating media.

Now, as to pieces of the liver, spleen, or kidneys—the two first can be operated on *in situ*, the last must be removed. The outside surface is seared over with a hot knife as before and the cut made in the same way; then with the cooled scalpel pieces as large as dice can be cut quickly out at a little distance from the cut surface and quickly placed in the sterilized bottles. If done correctly and quickly, and no adventitious germs are in these organs, the desired specific germ will develop in these pieces, as stated for the lymph glands. The liver is the only one of these organs that is liable of going astray for us, on account of the possibility of germs having been introduced into the gall-ducts by worm-parasites.

Let me say that uncontaminated blood serum can be very easily obtained and retained in a perfectly sterile condition for an indefinite period, and used for cultures in the thermostat at body-temperature by a proper technique. As I have never seen this fact mentioned, and it is invaluable as a cultivating medium, I will briefly describe the method which is invariably practised here for obtaining blood-serum. Naturally, everything used must have been thoroughly sterilized first.

The operation is made on the living animal in the same manner, as they are often used in veterinary colleges; except the jugular vein is used instead of the carotid artery. Cocaine is used to anæsthize the skin. A previously sterilized glass rod, to which is attached a sterilized rubber tube, is introduced into the jugular by the operator, who must have sterile hands. An assistant keeps the rubber tube from touching anything and pressed together. A ligature, or clamps, is put easily about the vein anterior to the place to be opened; a longitudinal cut is then made in the side of the outer wall, which has been first washed in the corrosive sublimate solution, the glass tube introduced and well bound; the ligature or clamp removed, and the blood at once allowed to flow into sterilized receptacles; we use preserve jars with metal caps, in which a smal hole, to be plugged with sterile cotton, has been cut a little larger than the end of the rubber tube which is introduced in it. *We have never yet had a fail in blood caught this way.*

The balance of the treatment is known to all. The receptacles are placed in an ice-chest for twenty-four hours, and the serum drawn off into tubes with sterile pipettes as usual. For fluid-serum cultures, the serum is at once placed in small flasks. We have flasks that have been in the laboaratory for months, in which the serum was obtained in this way, and they have always remained sterile.

There may be nothing new in all this, but I do not remember of any one's suggesting that blood-serum could be got in this way and never require sterilization, though as serum is now so often experimented with, I suppose it may be. At any rate, no harm is done, and describing the method may be useful to beginners.

One thing I desire again to emphasize, and that is the practicality of cultures made in the field in the above-named manner, for practitioners who desire to send material to laboratories for further investigation. *Very few utensils*, but the utmost exactness in detail is all that is necessary to attain success. This method is far more reliable than vacuum tubes, which cannot always be closed in a suitable manner, and much more practical than wrapping organs or large pieces thereof in sterilized cloths, soaked in some aseptic fluid, as I often do, however, so as to have all the controls possible, for one need not hurry to the laboratory, and farmers' houses are not always very suitable to work in, nor is it always agreeable. The majority of investigators knew little about making autopsies one hundred or more miles from their laboratory, and sometimes miles from a building with the wind blowing like a hurricane at sea, and the sun so hot they can scarcely bear it, and flies too thick to mention, or in winter so cold one can scarcely work, thus unprotected; but we have done much of that work, and have found the "blood" and "organ cultures" here described the most practical method open to us, and also that some few practitioners have been enabled to send very reliable materials for culture and experimental investigation when sent the sterilized bottles and directions how to use them.

### HOW TO MAKE SMEAR-PREPARATIONS FOR MICROSCOPICAL STUDY.

These directions are, as said, for the benefit of the every day practitioner who may desire to practice them a little as aids in diagnosis.

Having described how to expose the organs and the heart and take specimens from them, the rest is very simple.

The practitioner takes a wired-rod, if he has one, or his knitting-needle, as the case may be, and sterilizes the wire and the glass-rod for some way, or the needle in the same manner, and allows it to cool a moment and then dips it in the blood of the Ventricle, and spreads the blood out in a thin coating on the cover glass, and lets it dry in the air, and then in the flame, by passing the cover-glass, smear side up, two or three times quickly through the flame.

The liver, spleen, or kidneys, or lymph-glands are treated in the same way, except, if one desires a piece of the organ, a pair of small, slim, thin-pointed forceps are necessary, which must be sterilized in the same manner. Smears of such organs are nothing but a small piece thus taken out of the cut, at some distance from its surface, with the forceps, and rubbed out thin on the cover glass and dried in the air and in the flame in the way directed. They must not be held in the flame, for that will so destroy the germs so that they will not color well. The cover glasses are to be held with the forceps by the edge and passed rapidly through the flame.

As to coloring the smears, either a saturated solution of Fuchsin or Methylen Blue will do. But the Carbolic-Acid-Fuchsin solution is equal to the demands of the practitioner in nearly every possible direction he may desire, as it is also as reliable as any stain for tuburcle-baccilli.

R.

| | |
|---|---|
| 5 per cent. Solution Acid Corbol, - - | 100.00 |
| Alcohol (commercial), - - - - - | 10.00 |
| Fuchsin, - - - - - - - | 1.00 |

Filter before using.

To stain smears after being passed through the flame, simply hold the cover-glass, right side up, with the forceps, cover it with sterilized water, holding it level; add three or four drops of the above solution, and hold the cover a few moments; wash the whole off, and dry the glass with filtering paper. The oil of cedar, which is used for the lens, is also the best medium to examine such objects in. As to microscopes, a 1-16th oil immersion lens is the only reliable one, and, personally, I will not work with less than

1-20. Amplyfying Oculars are a fraud and a humbug. One wants to see what is under the eye, not a picture of a picture. Those who rely on Amplyfying Oculars see germs which bear as much resemblance to the original as reproduced photographs do.

THE BACILLUS OF SOUTHERN CATTLE PLAGUE DESCRIBED.

Before giving a detailed description of the discovery of the germs of this disease and the experiments which conclusively prove it to be the specific cause of the same, we will describe it:

In the former editions of this work it was said that "these organisms are neither to be classed with micrococci or bacilli; they are not round objects like the former, nor raised like the latter." They belong to that inter-mediate group to which, for convenience sake, patho-bacterialologists are beginning to give the name of "bacteria." This name is again being generally given up, and all micro-organism are now called "bacilli," in which the longitudinal diameter is double, or exceeds that of the transverse to any marked degree. Hence, we will now speak of the "Bacillus of Southern Cattle Plague" in the future.

FIG. I.

Diagramatic Representation of its Phases of Development.

The longitudinal diameter of these micro-organism is about twice that of the transverse. They are oiled. Their ends are rounded. They are so identically alike in size and shape with the Corn-stalk Disease germ that no one could possibly differentiate the one from the other by microscopic examination alone. They are somewhat shorter, less bacilliary, and perhaps a little thicker than the Swine Plague germ.

They color well in most of the Analine dyes, especially in Fuchsin and Methylen-blue. By suitable exposure to the tinction a distinct uncolored middle piece and sharply colored pole-ends can be easily demonstrated, but if colored too much, the middle piece or uncolored belt, becomes colored also, and then the germ is seen as a diffusively colored bacillus, or rod. This germ is motile, but it is far more lazy in its movements than either the germs of the Corn-stalk Disease or Swine Plague, (See Swine Plague Bulletin), though the movements are of the same nature. The illustrations given below (Fig. 2) presents as exact a picture of this organism as possible of its appearance in freshly prepared smears from the blood, liver, spleen, or lymph-glands, (for these germs can be demonstrated in those tissues, every and all assertions to the contrary), while the diagramic illustration (Fig. 1) illustrates the various phases through which the organism passes in its development. While the former (Figs. 2 and 3) forms also predominate in the smears made from cultivations, all the forms seen in Fig. 1 can also be seen in a properly prepared "hanging-drop" culture.

FIG. 2.
The Southern Cattle Plague Germ.

FIG. 3.

The Swine Plague Germ.

The morpho-vegetative phenomena of these bacilli is perhaps even better illustrated in the following cut :

FIG. 4.

Some people have doubted the accuracy of this description. The entire phenomena can all be seen by the prolonged comparative study of "hanging-drop" and dry stained cultures. To one unaccustomed or unproficient in this kind of work, however, the first appearance of a microscopic specimen of a culture of these germs, especially an aged one, would prove very puzzling indeed. In fact, such a person might easily conclude that his culture had become polluted by micrococci, or that some very much larger organism had invaded it. The latter is especially the case when cultures are made on material the surface of which has become quite dry and hard and the condensation fluid all evaporated. The mature form of this and similar organisms has been previously described and illustrated (Fig. 2), and resembles a microscopic lesion with its sides and ends so pointed as to leave the middle portion of the body untouched as we look down on it. This is the picture which the eye generally receives in smear preparations from the blood or organs and in freshly made cultures on fresh media. A more exact study of cultures, however, especially of "hanging-drops," will show that the above form is by no means the only one in which these bacilli present themselves to view.

Many individuals will be seen which while ovoid are much shorter, hence more oval, and the white belt will appear more like a spot in the middle of the object, not extending across it, (Figs. 1–1½). The non-colorable substance, when the germ is treated properly, is a secretion or product of the pole-ends, and in the above form only a small amount has been secreted, and not enough to completely separate or extend the ends. The whole organism is surrounded by a delicate capsule, which must be of the same material as the pole-ends, as it colors with them. The question at once arises, if the whole capsule colors, why do we only see evidence of the same on the sides and not in the portion directly under the eye of the observer, which appears uncolored. The whole capsule colors exactly alike, but is very thin, the substance separating the pole-ends does not color, and the capsule directly under the eye being so thin, we do not see the color, whereas looking at the sides, we look through more material, which thus gives us more color. The same is true of a thin piece of glass, looking directly through it, it is clear ; looking at it edgeways, it is blueish or greenish, as the case may be.

Again, we may see two or three of these organisms united together, all presenting the normal form of full maturity. (Fig. 1, No. 2). In general, however, they appear either singly or in pairs, though in certain media they have a tendency to form long segmented threads, as well as in some organs. This is applicable to the Swine-plague organism better than the one under consideration, so far as I have yet seen. In very old cultures numerous thin rods may also be seen which color diffusely, no uncolored substance being visible in many of them. (Fig. 1. No. 3). The same cultures are also extremely replete in cocci, which leads me to call this condition one of Coccio degeneration. That these are really morphic varieties of this organism is proven when we transfer such cultures to freshly made material, especially by plate-tests made on such material. This cocci-form represents the first stage in the development of these germs; they are the pole-ends separated from each other, as in dependent organisms. The first step is an elongation of the object. (Fig. 1. No. 4). Then one pole-end is dropped, giving a free coccus, and then the other becomes free. (Fig. 1, No. 5). In order to correctly observe these phenomena, the addition of a delicate amount of an Aqueous Methylen Blue solution to the fluid of the hanging-drop is necessary. When one of these coccio ends has been shed, the other acts like a plummet, and the other or free end is exposed to view in the drop, and we look down on a round colorless object enclosed in a delicate membrane which might be mistaken for a spore, but as the germ is motile and tumbles about, we soon see that such a conclusion would be erroneous. Germs in this condition, with one pole end in various positions, can be readily seen in dried or stained hanging-drop cultures. They cannot be seen in slides made from cultures grown on solid media.

As I said before, I do not know what becomes of the uncolored middle portion, unless it is a fluid secreted by the poles and contained within the capsule, which becomes free, as the pole ends are segmented and is also excluded from the germ into its surrouning media. I still think this substance, the uncolorable, represents the chemical products of the germs held in solution within the capsule, and that when it does color, we have either changed its character by the heat applied, or that in time the tincture penetrates it. That it has less affinity for the colors than the pole ends and capsule needs no discussion. Again, if we study very exactly, we see this substance first appear as a delicate, colorless paint in the coccaio, from the moment it begins to lengthen and assume an ovio form, and it increases in amount until the organism arrives at the full length, as represented in Fig. 1. Nos. 6 and 4. Other forms frequently seen are presented in Nos. 7, 8 and 9 of the same figure, and need no further description.

### GROWTH OF THE BACILLI OF SOUTHERN CATTLE PLAGUE IN AND ON DIFEERENT MEDIA.

The germs grow readily at normal temperature, and on the surface of potatoes, when those suturs are not too acid; the growth is generally of a delicate straw color, though, as I said in my previous papers, it sometimes assumes a reddish yellow color, when the colonies become old. The chromogenic proportions of germs, or any other productive property, can not always be depended on to differentiate or recognize them by; both media and the age of the germs outside the body cause a very marked difference in these directions. For instance, during the past winter we had some potatoes that had been buried in pits in the ground, to preserve them, which were so acid that none of the germs which generally developed freely on potatoes would grow on them at all. Then the potatoes themselves will effect the color of the colony as well as the time the germs have been cultivated ouside of the body.

On the white of eggs, when fresh cultures are used, these bacilli develop as a buff colony, with sharply defined edges, but this color becomes almost white when aged artificial cultures are used.

They do not fluidify gelatine, but a peculiarity in freshly derived cultures, which is gradually lost as they become old on agar-cultures, but retained in bouillon is that the line of development becomes dark brown, and is embraced in a cloud of dark brown substance of a lighter shade, which is a product of the germs after the cultures are three or four days old. When a very delicate wire is used, the small individual colonies may be seen along the whole line of the puncture if but few germs are on the wire. Quite a growth extends over the surface of the gelatine from the point of juncture, but as very thin coating.

Dr. Thomas Bowhill, of San Francisco, California, also investigated this disease, as will be noticed later on. Recently Dr. Bowhill was in Lincoln, on his way from England to California, and when in the Laboratory, looking over a lot of cultures, he at once picked out this germ in gelatine junctures, and remarked: "*That is just the way the germ grew in gelatine which I got from Texas Fever diseased cattle in California.*" I refrained from saying anything to him, and he could not have told from my labels what germ it was, as I have special marks for my cultures, for reasons of my own.

On plates the germ is small, round, greyish granulated-surface colonies when near the surface, but when deeper situated have a decided yellow tinge.

In Bouillon they grow very rapidly, clouding the material and in a few days numbers collect on the surface, forming a coating, but not a mycodlone, which the least jar disturbs, and they sink into the fluid to rise again, if left undisturbed.

On agar-agar they also dried up rapidly, but with nothing chorastic, the coating being thick and moist and of a grayish white color.

The bacilli have been time and again demonstrated in sections of the organs of peremptorily killed cattle, the pieces having been at once cut out and placed in absolute alcohol, so that first mortal change or invasion of the organs by any germs not present in the animal at the time killed is simply a matter of impossibility, though the contrary has been claimed in a certain quarter. As has been repeatedly said, *in order to obtain cultures and also the necessary pathological material for central examination, so far as my own work is concerned, in no single case since I began my investigations has such materialever been obtained from an already dead animal; every single one that I have personally used has been peremptorily slaughtered.* The already dead animals, and then only those in which first mortal changes had not occurred, have only been used for pathological studies of the lesions, though cultures have also been frequently taken from them for further evidence. Furthermore, though I have never taken pains to mention it, plate cultures have been resorted to with almost equal constancy. The only weakness, if such it is, in my earlier work, has been that being in search of specific germs only, and also having then been absolutely without any conveniencies, as well as without much money but my own, did use small pigs almost entirely instead of rabbits, and to-day, when I at once desire to prove a specific germ, I still resort primarily to the species in which the disease naturally occurs. We are better fixed now for small animals, as certain results will show, and in Chicago I had to resort to them often against my will, as the large domestic animals could not be used in the city, and thereby lost quite a number of very important observations, as results in small species of animals, or other than the naturally infested species, are more apt to be misleading than correct, as has been already shown elsewhere.

EVIDENCE THAT THE BACILLUS DESCRIBED IS THE SPECIFIC CAUSE OF THE SOUTHERN CATTLE PLAGUE.

We have first to describe the conditions in which we obtained this germ. As I have repeatedly asserted, the very first essential in the specific identity of any germ is that obtained, uncontaminated, in freshly killed or recently dead animals, afflicted with a specific disease, which has been, and is known for its specific clinical history and course, a disease over which there has not been and is not a doubt as to its actual clinical diagnosis. To my European colleagues I would say that no single disease in man or beast is more absolutely a unicum, more unmistakably easy of diagnosis than this southern cattle plague when it appears in northern cattle. As the southern cattle which generally come into the northern-western stock breeding States have almost universally come from Texas, the disease has acquired the name of "Texas Fever" among all farmers and breeders in the north, but for reasons given in another place the name which has been suggested as more appropriate is, "Southern Cattle Plague." The first thing then in the clinical diagnosis in this disease in northern cattle, is that the origin of it was in southern or Texas cattle. It does not take long to find that out, the farmers generally know that at once. The second is that we have no other such acutely deadly disease in northern cattle in this country. The third is its clinical phenomena, high fever, want of appetite, constipation and hæmaturia in the majority. The fourth is, that it is strictly local, and in all its history has never been known to cross a wire fence to other northern cattle in the adjoining field. A fifth is, the season of the year from May to November, in which it can occur. Severe frosts put an end to it. The pathological lesions are of less diagnostic importance with the exceptions of the diffuse engorgement of the kidneys, which will never be missed in the majority of the cases of natural infection having the usual course, especially in those which have had hæmaturia (see plate) ; in the more acute cases, especially where hæmaturia is wanting, the kidneys will not present this condition to any marked degree, though the initial stages of capillary engorgement will be present.

An error in diagnosis in northern cattle can be thus said to be an absolute impossibility to any one competent to judge and make suitable inquiries.

In the previous writing on this disease it was mentioned that I failed in obtaining cultures direct from the spleen of the slaughtered animals, and hence had recourse to the liver principally. It was also mentioned that the first case from which I obtained cultures was at Roca, Neb., in the summer of 1887. I now wish to correct both of those statements, and the reason that I did not mention this case in my former reports was that I had forgotten all about and misplaced my notes, and only found them on my return to Nebraska this time, as well as the slides made from cultures at the time. The cattle belonged to the Hon. John Fitzgerald, a very wealthy Irishman living in Lincoln. Dr. Thomas the best diagnostician of any veterinarian in the state, had charge of the outbreak as Assistant State Veterinarian.

I will give my notes just as they were written at the time and are still to be seen in the original record book :

"Oct. 6, 1886. Dr. Thomas brought a piece of the spleen of a heifer, said to have died of Texas fever, to the laboratory. (It was late at the time, and we had to use lamp-light.) Some small objects, short and round-ended, were to be seen in spleen sinuses. *Cultures* gave the same object in pure condition. A mouse was at once inoculated with a small amount of splenic pulp. It died in twenty-four hours. Abdominal walls stained yellow-red, as well as pericardium. Spleen immensely swollen. Cultures from the spleen of the mouse gave peculiar ovoid bacteria about the size of the swine plague germ. They grew rapidly on agar in a pearly-whitish color. In gelatine they grew much like the swine plague germ, and extends over the surface from the point of puncture. Does not fluidify gelatine. It seems, I then inoculated a rabbit with 1 ccm of a twenty-four hours bouillon culture, and that it took five days to kill it, and that it died of septicæmia." As mentioned, this case had entirely slipped my mind when I wrote my reports, which is not to be wondered at considering the continual state of irritation I was kept in during my first period in this State. It may be asked why I did not go further in my investigations at that time? I could not possibly. I was paying nearly all the expenses of my work, and had neither assistant nor servant, even washing the floor of my room, taking care of my animals, and doing all a servant's work, besides I was giving all my attention to swine plague, and out in the country most of the time. I have still slides of the cultures made at that time, and considering the undoubted character of the diagnosis and the results here detailed, I do not think any one can raise a reasonable question as to the accuracy of the work, hurried and imperfect as it was. It may as well be mentioned that in company with Dr. Thomas and the State Cattle Commission that I also visited the sick herd. As it was Mr. Fitzgerald's business and late in the season, and as the sick cattle were closely fenced, I also remember that they were quietly left in the hands of the owner and his veterinarian.

This case then shows this, that a germ growing like and microscopically identical with the one found later on, and one at present in my laboratory from a still more interesting source in pure cultures, was obtained at that time from the spleen of an animal which died of Texas fever, and that there was no error in the diagnosis.

Another very corroborative point, which I noticed in my later investigations and have confirmed the past winter many times, was the great resistance of rabbits to this germ, it requiring exceptionally large doses to kill them, while mice succumb very quickly.

At this time my esteemed friend, Dr. Thomas Bowhill, was with me spending all his spare time working in the laboratory, though practising his profession also. He afterwards went to San Francisco, Cal., and in the *Veterinary Journal*, London, 1890, Vol. XXXI, p. 1, publishes some investigations on the same disease in California, from which I quote the following :

"The first outbreak occurred in October, 1888, and was a very serious affair, one rancher losing 700 out of a herd of 1,200, and this mortality in from three to four weeks. I made six full autopsies and cut open about fifty other carcases simply to observe the condition of the liver and spleen. In November and December, 1888, I investigated the second outbreak, and made four full autopsies. In February, 1889, I made my third investigation, and performed ten autopsies. It was during this outbreak that I obtained the first cultivations in gelatine and on agar. I could not then define any distinct organisms, as my highest power was only an ⅛ of an inch.

"I made my last examination at a dairy, within the city limits of San Francisco, in October, 1889, and as I had now obtained a $\frac{1}{12}$ oil immersion lens I was able to make a thorough examination of cover-glass specimens and cultivations.

"Billings, in his work, claims the cause to be an ovoid-belted organism, and gives experimental inoculations in support of his assertion.

"Dr. Smith says he inoculated rabbits and they remained healthy, but again Billings says, p. 125, he found rabbits to possess natural immunity to at least ½ ccm. injection.

"In October, 1889, I obtained some cultivations on sterilized gelatine from the liver and spleen of a well marked case. Those cultures from the liver developed splendidly, but those from the spleen remained negative. The cultures from the liver did not liquify gelatine, and preserved a yellowish color at first, becoming darker as they grew older. I also obtained two cultures from the liver on white of eggs as described by Dr, Billings in his work.

"In cover-glass specimens, from the cultures as well as from the liver, spleen, and gall-bladder, an organism similar to the one described by Billings was observed staining most distinctly in fuchsin. A mouse inoculated with two drops of a bouillon culture died in less than twenty-four hours. A cultivation was obtained from the liver of this mouse, which developed exactly the same germ as the original one."

I will say that I have slides from Dr. Bowhill's cultures as well as sections stained by himself from the organs of the cattle, from which he obtained his cultures, and the germ, as described by him, is in them all, and that, microscopically, it exactly corresponds with my own material.

Attention might be also called to the fact of the extreme mildness of the climate in California in comparison to this section of the country, which accounts for the disease occurring there in months it is never seen here, on account of the cold weather.

As said before, there is no possibility of a mistake having been made in diagnosis, as we have no other cattle disease in this country with the same peculiar clinical history and malignantly fatal course.

We will now return to my own investigations, taking first those cases from which I obtained cultures and later pathological material and demonstrated the germ in the same, and also notice material sent in which I demonstrated the germ in one way or the other.

THE OUTBREAK AT ROCA, NEB., SEPTEMBER, 1887.

Fifty-eight head of graded southern cattle were bought by a Mr. F., of this locality, on the 28th of June, of a live-stock commission house at Kansas City, Mo. On the first day of July they were placed in a pasture with a lot of Nebraska cattle, and on the 29th of August two of the northern cattle died, and another on the following day. Having been informed of this outbreak I immediately visited it in company with Dr. Thomas.

Autopsy 1—Native steer two years old. Before killing the animal the temperature was taken and found to be 41 degrees C. It was in fairly good condition ; had been ill two days, body covered with ticks ; visible mucosæ injected and of a yellowish color. Some appetite. Flanks tucked up and fallen in ; hair bristling ; movements weak and uncertain, especially those of the hind legs. The animal was knocked in the head and its throat cut. Blood quite thin, and while it coagulated, the process proceeded very slowly ; it was of a claret-red color, and presented the same when running over the fingers, in fact it seemed more like a red water than blood, not having the usual viscidity of that fluid. On skinning the animal the subcutaneous adipose tisssue was somewhat atrophied and of an abnormally yellow color, the abdominal aponeurosis being also tinged with the same color. The muscles of the animal were of a greyish-red color. On opening the abdominal cavity the two first things which struck the eye were the diffuse pinkish-redness of the outside covering of the small intestine, and the enlarged and prominent spleen, which was about double its natural size and weighed five pounds ; contents rich in blood, somewhat softened, and of a dead red-black color. The stomach and intestines were next removed. The first stomach was two-thirds full of grass with considerable fluid admixed ; the contents of the second stomach were still more fluid, while the third was distended but not over hard ; the fourth stomach was empty, its mucosa was swollen and reddened with small hæmorrhagic centres here and there ; it was also covered with a thick viscid coating, which was closely attached to underlying tissues. Contents of small intestine semi-fluid, and not sufficient in amount to fill the tube ; that of the large intestine was of a pultaceous character ; the mucosa of the entire tract was swollen and covered with an adhesive viscid coating characteristic of catarrhal conditions ; that of the small intestine, especially the anterior two-thirds, was of a bright-yellow color, being deeply stained with gall ; the posterior portion was of a more deep-red color, otherwise in same condition. Liver swollen, and of yellowish-grey-red color ; the capillary gall ducts were so distended that they could be easily seen with the naked eye in many places ; the acini were distended. Kidneys swollen and of an intense bluish-red color, though the swollen urinary tubes could be seen through it as greyish-white striæ ; the mucosa and fat of the pelvis of the kidney was of a bright-yellow color. Bladder about one-half full of a claret-red fluid, which had a specific gravity of 115. The abdominal lymph glands were much swollen and moist ; in many of them there were hæmorrhagic centres, while others were of a diffuse dark, bluish-red color. A small quantity of effusion in thoracic cavity. Pleuræ swollen and of a yellowish color, with a few hæmorrhagic points here and there. Fat around heart stained yellow ; pericardial sac contained about a tumbler of yellowish colored fluid ; its muscle was soft and friable, anæmic and of a yellowish grey-red color. Bronchial lymph-glands much swollen and presented similar conditions to those of abdominal cavity. Anterior and posterior extremities of both lungs presented a mottled and dark-red color, and were the seat of numerous centres of lobular pneumonia, cut surface same as outside, but more lustrous and œdematous; inter-lobular vessels engorged; in some the blood was coagulated; these were a secondary complication due to disturbances in the circulation. Mucosa of bronchial tubes swollen, tinged yellow, marked by a few petechiæ; the smallest bronchi, especially those in the diseased portions of the lungs, contained a watery fluid.

Autopsy 2.—This animal was in the same herd, and killed immediately after the necropsy was completed on the previous one. Native grade steer, two years old. As usual it had withdrawn itself from the herd, and stood, near a run, alone by itself. On stirring it up, its movements were tottering and very feeble, unless hazed, when it still had strength enough to do some pretty smart running. A close examination of the animal was impossible. It frequently tried to pass manure but was unsuccessful, except in very small quantities. Somewhat emaciated, hair rough, eyes had an anxious expression. Although evidently a very sick animal the owner did not feel like killing it, so that we purchased it for the sum of ten dollars. Shot the animal. Immediately on falling took its temperature, which was 42.40 degrees C. Visible mucosæ stained yellow, especially prominent in the conjunctivæ.

No external marking worthy of note. Paniculus adiposus considerably atrophied; blood of the peculiar log-wood color and character found in this disease. Coagulated and oxidized slowly on exposure to the air. Peritoneal cavity contained about six quarts of an amber-colored fluid. Costal peritoneum swollen and clouded, and marked by numerous petechiæ spots. Serosa of small intestines of a diffuse pinkish-red color; some few small hæmorrhagic centres present; that of large intestines clouded, swollen, grayish-red in color, with numerous petechiæ here and there. Spleen exceeding swollen, contents semi-fluid, weight four pounds, and covered with vegetations in various places. Gall-bladder distended and full of a greenish-yellow fluid. Cut surface of liver somewhat anæmic; acini very much swollen, general color being yellowish-grey-red—intra-acinous veins invisible; gall capillaries presented a beautifully injected condition. Kidneys swollen; capsule non-adherent; external and cut surface of cortex being of a diffuse logwood red color; the medullary substance also a diffuse red, but of a lighter shade; mucosa of pelvis swollen and replete in hæmorrhagic centres of different dimensions. The bladder contained about a quart of claret-colored fluid; its mucosa was much swollen, vessels injected; many small hæmorrhagic centres present. Nothing need be said of the first three stomachs, but the fourth offered a fine picture of the lesions seen in this disease. It was empty ; mucosa very much swollen and of a very intense, deep, but not dark, pink-red color, the diffuseness of which was interrupted in many places by hæmorrhagic centres, and in others were spots covered with a greyish-yellow material, somewhat dry, and which, on being removed, exposed an ulcerated surface, which was below the line of the surrounding tissues ; the edges of these ulcerations were irregular and swollen ; the balance of the mucosa was covered with a viscid adherent coating ; vessels of sub-mucosa intensely engorged ; numerous hæmorrhages being also present in these tissues ; towards the pylorus the mucosa was of a deep yellow tinge, which extended throughout the duodenum into the jejumum ; contents of the latter semi-fluid in character and dirty yellow in color ; mucosa swollen, presenting similar conditions to those described in the stomach.

Large intestines ; contents semi-fluid and of a dirty yellowish-green color ; mucosa swollen and of a light pink color ; numerous hæmorrhagic centres here and there, which become more profuse and diffuse, as well in extense as the rectum was approached ; towards the anus the entire mucosa was a dense dark-red color and very much swollen ; the custs of the folds were marked by small centres covered with a dry yellowish-gray material. No effusion in thoracic cavity ; lungs comparatively normal ; myocardium opaque, anæmic, yellowish-gray-red in color and friable. Lymph glands of entire body swollen, œdematous, and of a diffuse pink-red color.

It was from these two autopsies that I first obtained the germ of the southern cattle plague. The method previously described of obtaining cultures direct from the blood had not then been much used by me, and its reliability not known, as it is now, so being eighteen miles from the laboratory, a large piece of the liver from each animal was at once cut out as soon as that organ was exposed and wrapped in a towel saturated with a strong enough solution of carbolic acid to corrode the outside, and the whole put into a glass preserve jar, out of which the carbolic acid solution had been poured after taking out the cloth. Objections have been made to this method, but it has worked, and still works, very reliably in my hands when the material is at once taken to the laboratory and cultures made, and I do not think such objections justifiable. Attention has been previously called to the fact that tubes cannot always be used in the field on account of the strong wind generally blowing here, and the extreme heat and flies in summer are also an obstruction. In this case, the nearest building was five miles. On completing the examinations, we at once drove back to Lincoln and were at the laboratory in an hour and a half; tubes and bouillon flasks were at once inoculated, and the ground squirrels which I used in making these studies, a most convenient animal when we can get them here in the west. Plates were also made. The organism herein described was found present in all the cultures, and killed the squirrels, and was found in their blood.

*Southern cattle plague produced by inoculation with pure cultures obtained above.*

As soon as the purity of the cultures had been determined by plates and microscopical examinations it was decided to at once test the specific value of the bacillus obtained by inoculating some healthy cattle. This was done on Saturday, September 10, 1887, a red cow and a five months old black steer receiving 10 ccms. subcutaneously in the dew-lap. The cow was a very wild animal and it was impossible to catch and handle her again for examination, but she fell off in her feed and condition, became constipated, but finally recovered.

The black steer was off its feed, hair bristling; the animal stood much by itself; respirations accelerated, but not labored; temperature elevated. First observed to be unwell on Wednesday, the 14th; temperature, 4 P. M., 42.50 degrees C.; Thursday, 15th, temperature, 4 P. M., 42.25 degrees C.; Friday, 16th, 41.50 degrees, 9 A. M.

*Necroscopical notes on black steer inoculated with pure cultures obtained from the Roca cattle.*

Seeing that this animal might recover, and desiring to observe the lesions produced by the inoculation, and also to oblige my friend, Dr. C. C. Lyford, of Minneapolis, who was visiting me and had never seen an autopsy in a case of the southern cattle plague, it was determined to kill the animal. The fact that the same micro-organism as that injected was obtained from its tissues in pure cultures, and a comparison of the following necroscopical notes, with those of the original cattle, and others following from a steer from another outbreak, should convince any unprejudiced person of the reliability of these investigations and that southern cattle plague is caused by specific bacteria. Blood of the peculiar color and lac consistency, which is more or less characteristic of this disease. (Some freshly flowing blood was caught in a sterilized bottle as it spurted from a cut artery and the same micro-organisms afterwards found in it). Paniculus adiposus, somewhat atrophied and of a decidedly yellow color; costal peritoneum and omentum, as well as the serosa of the large intestine were of the same color, interrupted by numerous petechiæ. Small intestine of a general diffuse pink-red color, variegated by engorged vessels and a few petechiæ; blood-vessels of mesenterium engorged; lymph glands swollen and odematous; interstitial and sub-capsular vessels injected. Other lymph glands the same. Abdominal cavity contained about two quarts of straw-colored fluid. Spleen swollen, full of blood, and soft, weight two and a quarter pounds. It must not be forgotten that this animal was but five months old. Liver swollen; edges rounded; central vessels of acini invisible, while the interacinous vessels were injected; each acinus was most beautifully decorated by delicate lines of a bright yellow color, which represented distended interacinous gall-ducts. Gall bladder distended. As I believe this pathological injection of the gall capillaries to be, perhaps, pathognomonic of this peculiar infectious disease in cattle, I desire to call attention to a necroscopical remark on the same subject made by the government investigators in connection with the cattle which they claim to have killed by placing on them young ticks artificially hatched from collected ova, they say: "Liver weighs about twelve pounds; enlarged; yellowish in sections. Complete injection of the intra-lobular bile capillaries." Report Bureau, A. I., 1889–1890, p. 98.

It is remarkable that both ticks and germs should produce the same pathognomonic lesion in the same disease, and yet in their cases no germ exists; and, none was looked for by their own records.

Here is a very weighty piece of testimony against the young-tick-protozoön inoculation theory of the Government, given by its investigators:

"These brief notes demonstrate that Texas fever can be produced by placing young ticks on cattle, and that the disease cannot be due to any abstraction of blood, for the ticks were still quite small, and had scarcely begun to draw blood on a large scale." (Then from some had not "any blood" been abstracted. How then could so many protozoa have gotten into the blood of the cattle as to kill them under those circumstances? And, as has been said before, if the ticks drew blood, and the young ticks in this case having been artificially hatched from collected ova, and the young ticks never having been in any relation with diseased cattle or according to these investigators, and the possibility of the same being absolutely barred, how then can the protozoa have gotten from the young ticks into the blood of the cattle and caused disease under those circumstances? The whole order of the tick economy must have been reversed. Instead of sucking in blood, they must have stuck their proboces into the skin and puked out protozoa. No other way of protozoa-inoculation is possible according to the Government theory of tick transmission.)

Kidneys swollen; outside surface most beautifully marked by the injected condition of the inter-tubular vessels and presented as fine a picture of natural injection as could be desired, as well as illustrates the earliest stage of that general renal engorgement which is represented by the intensely swollen, bluished-red kidney so often seen in this disease under conditions of natural infection.

Stomachs partly full. Mucosa of the fourth stomach very much swollen and of a diffuse dark-pink-red color, interrupted by numerous engorged vessels, ecchymoses and diffused hemorrhagic centres. Mucosa of duodenum and jejunum swollen and of a yellowish-red color: Peyer's patches and solitary follicles much swollen; large vessels engorged; occasional hemorrhages in the mucosa; contents semi-fluid. The yellow-staining of the mucosa of the anterior part of the small intestine was lost in the ileum, though here the membrane was also swollen, but of a diffuse pink-red color. Mucosa of large intestine swollen and of a delicate diffuse pinkish-red color; some engorged vessels present; this condition increased in intensity from the beginning of the rectum towards the anus, where the mucosa was intensely swollen and of a dark-pink-red color, with small hemorrhagic centres, especially on the crests of the rugæ; contents more and more solid until they become quite hard in the posterior portion of the rectum. Nothing abnormal in the thoracic cavity except that the lungs were slightly hyperæmic; the bronchial lymph-glands were swollen, red and œdematous. Myocordium opaque, anæmic, yellowish-red in color and somewhat soft. Mucosa of trachea swollen, vessels injected, some few petechiæ centres. Bladder half-full of straw colored urine; vessels of mucosa somewhat engorged.

Smear preparations from the blood and organs gave the same bacteria injection, as well as did the cultures from the blood and organs which were carefully compared with the originals.

In comparing the virulence of bouillon-cultures from this calf in ground-squirrels, the same dose was found to be less actually fatal than that of the original material. This I do not consider at all remarkable, as the land of the southern states and the bovine organism is the natural habitat of this parasite.

Reported examinations of sections of the organ of all the animals mentioned here have since been made and always with positive results. Again, in all cases, the pieces from which sections were to be made were cut out ere the animal was cold and immediately placed in absolute alcohol.

It is impossible for me to conceive how any man could more exactly fulfil every condition of exact investigation more completely than it has been done above. The most rigid examination of the lesions induced in the inoculated animal with those in the natural diseased cattle must show the most exact reproduction in every particular, except that the circulation disturbances in the kidneys of the young inoculated animal were not so advanced as in those from which the germs were obtained, but the "mahogany" kidney, while very common, is not an absolute necessary phenomenon in this disease.

It must be mentioned that there were no ticks on or about the inoculated cattle, and no diseased cattle nearer than those in Roca, eighteen miles away, so far as known.

Autopsy 4.—It would seem as if further evidence were absolutely unnecessary, but as I hope in this edition of these investigations to so place the bacterial etiology of the southern cattle plague before the world, that no one will dare question its reliability, and thus have prepared the way for a more brief description of the disease. This animal is from the second outbreak at Tekomah, Neb., the latter part of September, 1887, and from this animal's blood and organs pure cultures of the same micro-organism as in the others were obtained and subjected to every necessary control and test. Small pieces of the organs were also placed in absolute alcohol while the animal was still warm.

It may be as well that I mention here that I did not obtain cultures from the first Tekomah outbreak, having tried in vain to inoculate tubes in a hurricane, with a hot, dry, dusty wind blowing, and obtained a complete set of the fungus flora rather than germs. The tissues, however, were properly placed in alcohol, and the same germ can be seen in them by any one competent to examine them.

Now to our steer. Red and white steer, two years old, very good condition. Temperature 42 degrees C. Respirations rapid and distressed. Eyes fixed and becoming glassy. Pulse very rapid and weak.

Cut its throat. Blood of a peculiar reddish color, having more the character and appearance of some coloring fluids than normal blood. It coagulated but slowly on exposure to the atmosphere and did not become scarlet-red as quickly as most blood does under the circumstances. Paniculus adiposus well preserved Superficial inguinal glands intensely swollen and of a diffuse reddish-grey color ; but surface moist and glistening ; a red, watery fluid exuded on pressure. The other lymph glands in the body were in the same condition ; those of the mesenterium being the most excessively so, and replete in blood. The abdominal cavity contained about a quart of a yellowish-colored fluid. Peritoneum also stained with the same color. The outside of the small intestines presented a bright diffuse pink-red color, while that of the larger was more greyish-red. The spleen was much swollen, and presented a peculiar variegated appearance, the superficial veins being intensely engorged, and the tuberculæ showing through the capsule. Weight, six pounds. It was twenty-one inches long, eight wide, and three thick in the middle portion, which was much more swollen than at either end. When cut, it was found softened and completely engorged with blood which flowed out of the substance of the organ.

I did not stop to remove the liver, but at once cut out a piece on opening the animal and wrapped it in three clean napkins that had been soaked in five per cent. carbolic acid solution for over a week. The whole organ was very much swollen, its edges thick and round ; gall bladder distended and full of a greenish-yellow gall ; cross sections demonstrated the liver to be quite full of blood, inter-acinous vessels engorged, acini destended and yellowish grey in color ; gall capillaries markedly injected, and could be seen as delicate hair-like lines of a yellow color working the limits of each acinus. Kidneys, intensely swollen, the left one weighing two pounds. Fatty capsule the seat of more or less extensive hæmorrhages ; capsule non-adherent. Outer surface of a diffuse, dense logwood, red, some of cut surface ; nudually surface could not be distinguished from the corsical so far as any difference in color went ; mucosa of pelais swollen and the seat of numerous hæmorrhogic centres ; cavity contained a quantity of coagulated blood which was attached to the mucosa. *Blader*, two-thirds full of claret-colored urine ; mucosa swollen ; vessels engorged with numerous diffuse and small circumscried hæmorrhagic centres scatted through its substance.

*Stomachs.* First three comparatively normal. The fourth contained a small quantity of gall-stained, greenish-yellow material. Mucosa intensely swollen and of a diffuse livid pink color interrupted by the engorgement of the larger vessels and numerous hæmorrhagic centres of various size and contour ; aside from these were numerous ended spots with swollen thickened edges, the base being of a reddish-gray color ; the entire mucosa was covered with a viscid-catarrhal coating which was of a yellowish color in the pyloric region. Mucosa of small intestine much swollen and of an intense yellowish-red color, interrupted by numerous hæmorrhagic centres. Solitary follicles and Peyer's patches swollen, latter, markedly. Contents semi-fluid and of a dirty yellowish-green color. Mucosa of large intestine still more swollen and of a deep-red color ; hæmorrhages more extensive and frequent ; these conditions increased towards and in the rectum where the swollen folds were marked ; towards the anus the hæmorrhagic engorgement of the mucosa were still more extensive, being of a diffuse-pink-red color. Contents pultaceous and of a yellowish color, stained with blood on the surface.

Thoracic cavity free from effusion. Pericardial sac held about two tablespoons of reddish colored fluid and covered with petechia. Myocardium, opaque, anæmic, friable. Bronchial lymph-glands like others. Lungs normal ; mucosa of trachea and bronchi somewhat swollen, numerous petechiæ. Covering glasses were at once smeared from the first blood caught in a sterilized bottle also from the liver and cultures made on Agar on return to town. In the blood, and also the covering glasses preferred from the liver the same germs here described were present in great numbers. Cultures of the same also developed.

The observations, thus far recorded, were made in 1887. They were again confirmed in every detail, with the exception of cattle inoculations by autopsies and examinations of slaughtered animals in outbreaks at Gibbon, Neb., in August, and at Plattsmouth, Neb., in September, 1888. About the same time, my colleague, Dr. R. J. Withers, in Chicago, sent me some carefully collected fresh and alcoholic material from cattle that had perished in certain experiments then being carried on at the stock yards in Chicago by the Illinois Live Stock Commission. By means of plate culture and the squirrels, the same germ was again isolated from this material and is present in the alcoholic material.

To sum up them : One and the same micro-organism has been isolated in pure cultures, found in serum from the blood and organs, and in alcoholic material in an outbreak of the southern cattle plague at Roca and at Tekomah in 1887, in the same manner in outbreaks at Plattsmouth and Gibbon, Nebraska, in 1888, and also in material sent from the experiments made by the Illinois

Live Stock Commission the same year, and the disease has been produced in a young steer by the subcutaneous inoculation of pure cultures of this same germ, and pure cultures of the bacillus thus found ; again obtained from the blood and organs of the inoculated steer, and demonstrated in alcoholic specimens of the same. The same germ has also been demonstrated in all the details (except the cattle experiment) by Dr. Thomas Bowhill in San Francisco, from the blood and tissues of sick cattle.

Is the bacillus of anthrax, of tuberculosis, of glanders, any more conclusively proven than this of the southern cattle plague ?

In the face of such evidence as that, however, we find the Chief of the Bureau of Animal Industry, an individual absolutely without credit as an investigator, and an officer of the Government of the United States, telling the world that :

" I am now prepared to say that Texas fever is not a bacterial disease. All the illustrations which have been published showing preperations of the blood from Texas fever animals swarming with bacteria, and sections of tissues showing the same micro-organisms, are misleading and one of no value to the student of this disease. The blood and tissues do not present such appearances when properly examined."

The evidence is all here. The bureaucratic head admits that illustrations of blood swarming with bacteria and " sections of tissues showing the same micro-organisms " have been made. Such things then exist. Furthermore, they have had them and seen them in Washington. They do not dare deny their existence, or that they were obtained from cattle suffering from Texas fever. All that they can say is that " the blood and tissues do not present such appearances when properly examined." Who is to judge of the " proper " in this matter. Who gives the verdict? A person notoriously known not to have done an iota of scientific investigation in his life, or one in whose work the most searching reinvestigation cannot and will not find a single material error ? Error, so far as the bacilli of the Southern cattle plague is concerned, is a thing not to be thought of for a moment in these investigations. The evidence of the autopsies, made on animals purposely killed, and when the flesh was still quivering, " blood-cultures " made from the blood caught directly in stirilized vessels (this is how the swarms of the " same bacteria " as demonstrated in the sections were produced) controlled by cultures from organs made in tubes within an hour during which time the piece of organ was enclosed in cloths saturated with 5 per cent. carbolic acid ; sections of organs made from small pieces cut out while the organ was still warm and the heart often beating and placed in absolute alcohol. Pure cultures obtained and the same disease produced by inoculation and cultures again obtained and all this confirmed by other outbreaks and material collected with the same rigid exactness and finally confirmation by Dr. Bowhill. What disease of the human brain could lead any person to such imbecilic assertions as the efforts of the department to strengthen itself in the eyes of the "fool farmers" by trying to shake our work by misrepresentation? It is my unpleasant duty to still further open up the ulcer of political corruption in the Farmers' Department of the Government of the United States.

*To be Continued.*

## Figures and Tables

**Fig. 1. f1:**
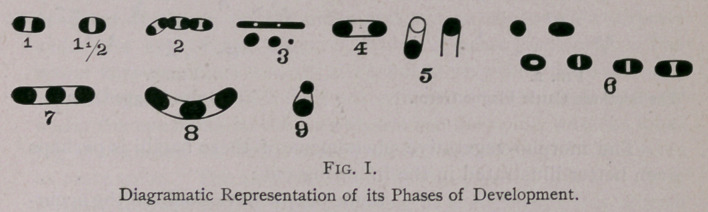


**Fig. 2. f2:**
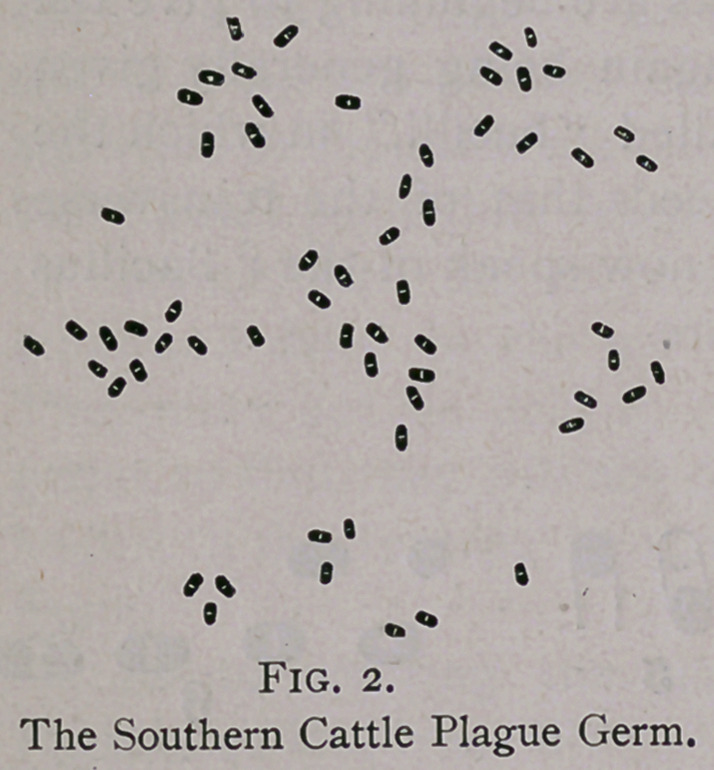


**Fig. 3. f3:**
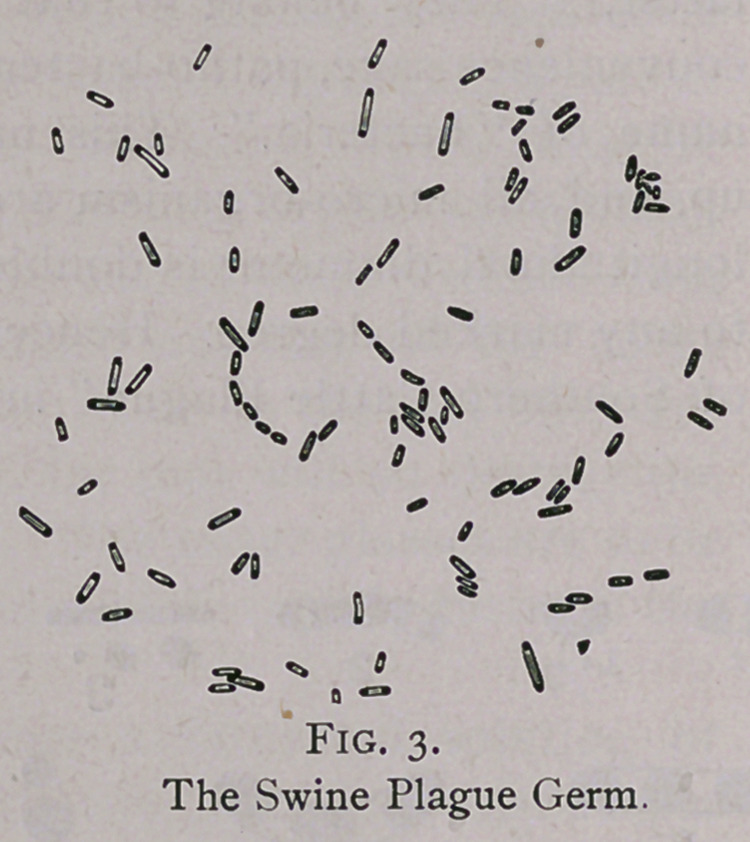


**Fig. 4. f4:**